# Supplementation with *Achyrocline satureioides* Inflorescence Extracts to Pregnant and Breastfeeding Rats Induces Tissue-Specific Changes in Enzymatic Activity and Lower Neonatal Survival

**DOI:** 10.3390/biomedicines5030053

**Published:** 2017-08-29

**Authors:** Karla Suzana Moresco, Alexandre Kleber Silveira, Carlos Eduardo Schnorr, Fares Zeidán-Chuliá, Rafael Calixto Bortolin, Leonardo da Silva Bittencourt, Moara Mingori, Luana Heimfarth, Thallita Kelly Rabelo, Maurilio da Silva Morrone, Juliana Poglia Carini, Daniel Pens Gelain, Valquiria Linck Bassani, José Cláudio Fonseca Moreira

**Affiliations:** 1 Departamento de Bioquímica, Universidade Federal do Rio Grande do Sul (UFRGS), 90035-000 Porto Alegre, RS, Brazil; alexandre.k.silveira@gmail.com (A.K.S.); ceschnorr@gmail.com (C.E.S.); fzchulia.biomed@gmail.com (F.Z.-C.); 00159410@ufrgs.br (R.C.B.); lsbittencourt@gmail.com (L.d.S.B.); moara.rmingori@gmail.com (M.M.); luanaheimfarth@gmail.com (L.H.); talitabioq@gmail.com (T.K.R.); maurilio.bio@gmail.com (M.d.S.M.); dgelain@yahoo.com.br (D.P.G.); 00006866@ufrgs.br (J.C.F.M.); 2 Faculdade de Ciências da Saúde, Centro Universitário Ritter dos Reis (UniRitter), 90840-440 Porto Alegre, RS, Brazil; 3 Faculdade de Farmácia, Universidade Federal do Rio Grande do Sul (UFRGS), 90035-000 Porto Alegre, RS, Brazil; juliana.carini@ufjf.edu (J.P.C.); valquiria.bassani@ufrgs.br (V.L.B.)

**Keywords:** *Achyrocline satureioides*, toxicity, gestation, neonatal mortality

## Abstract

*Achyrocline satureioides* (AS, family Asteraceae) is a plant widely used in traditional medicine for stomach, digestive, and gastrointestinal disorders during pregnancy. Studies regarding the indiscriminate use of plant infusions during pregnancy are limited. Recent reports have shown that chronic flavonoid supplementation induces toxicity in vivo and raises the mortality rates of healthy subjects. Therefore, we investigated whether supplementation of pregnant and lactating Wistar rats with two AS inflorescence extracts, consisting of an aqueous (AQ) extract similar to a tea (47 mg·kg^−1^·day) and a hydroethanolic (HA) extract (35 mg·kg^−1^·day^−1^) with a higher flavonoid content, could induce redox-related side effects. Total reactive antioxidant potential (TRAP), thiobarbituric reactive species (TBARS), and total reduced thiol (SH) content were evaluated. Superoxide dismutase (SOD) and catalase (CAT) activities were additionally quantified. Our data suggest that both AQ and HA of AS inflorescence extracts may induce symptoms of toxicity in concentrations of (47 mg·kg^−1^·day) and (35 mg·kg^−1^·day^−1^), respectively, in mothers regarding the delivery index and further decrease of neonatal survival. Of note, significant tissue-specific changes in maternal (liver, kidney, heart, and hippocampus) and pups (liver and kidney) biochemical oxidative parameters were observed. Our findings provide evidence that may support the need to control supplementation with the AQ of AS inflorescence extracts during gestation due to potential toxicity in vivo, which might be related, at least in part, to changes in tissue-specific redox homeostasis and enzymatic activity.

## 1. Introduction

Medicinal plants, which have contributed extensively to the development of modern medicine, have been used for centuries to treat several diseases and continue to play a significant role in drug discovery [1]. During recent decades, interest in identifying metabolites from plants that exert beneficial effects on human health has increased. Among these metabolites, antioxidants or free radical scavengers have received particular attention for their pharmacological potential [2]. *Achyrocline satureioides* (AS, family Asteraceae), popularly known as “marcela”, is one of the 25 *Achyrocline* spp. described in the Brazilian territory [2]. AS is a medium-sized aromatic annual herb, commonly found in tropical and subtropical America [3]. This plant is collected before sunrise, and the naturally dried flowers are used throughout the year to treat several gastrointestinal disorders [4].

AS is considered a promising medicinal plant, which has been used for a long time in folk medicine, and is also a designated official vegetable drug in the Brazilian Pharmacopeia [3]. In fact, previous in vivo and in vitro studies have provided evidence supporting the traditional use of AS as an anti-inflammatory, hepatoprotective, antioxidant, immunomodulatory, antimicrobial, antitumoral, and photoprotective agent [3,5,6,7]. Furthermore, in vitro analysis have shown that AS is cytotoxic at higher concentrations (588–653 μg·mL^−1^) [8]. Investigations of chemical composition revealed the flavonoids quercetin, 3-*O*-methylquercetin, and luteolin as the main compounds in AS inflorescence extracts [9]. These isolated compounds have demonstrated, in vitro, some pharmacological activities, such as scavenging of reactive oxygen species (ROS) [4,5,9]. This scavenger property is very important considering that ROS and other reactive species have been implicated in the pathology of over 100 human diseases [10].

The potential health benefits and general assumption that natural products are safe have increased the consumption of dietary flavonoid-based supplements by the general population, including pregnant women [11]. Pregnancy is a condition associated with large physiological changes resulting in numerous pregnancy-related symptoms, including nausea, vomiting, constipation, and heartburn [12]. Many women resort to the use of medicinal plants to alleviate these symptoms and one of the most widely consumed is *A. satureoides* infusion [3].

Herbal products are preferred over prescription medications in pregnant women because they are believed to be safer for the fetus than modern medicines are [13]. However, unlike conventional drugs, the use of herbal medications is not strictly regulated and, unfortunately, the potentially toxic effects of excessive flavonoid intake are largely ignored [14]. At higher doses, flavonoids may act as mutagens, pro-oxidants that generate free radicals, and inhibitors of key enzymes in hormone metabolism, such as kinases [15,16] and topoisomeras [17,18]. Concentrations of 50 µM of quercetin can inhibit the mitochondrial respiratory chain [19]. Unrepaired or misrepaired oxidative DNA damage can result in DNA strand breaks and mutations [20] that may lead to irreversible preneoplastic lesions. Furthermore, high intakes of these compounds may potentiate other deleterious effects due to their diverse pharmacological properties, which may alter drug and amino acid metabolism, modulate the activity of environmental genotoxicants, and alter the activity of other key metabolizing enzymes [14].

Flavonoids also act as powerful antioxidants in vitro and in vivo by scavenging diverse reactive oxygen species (ROS) or inhibiting their formation [21]. In vitro studies also showed that treatment with components of energy drinks (caffeine, taurine, and guarana) with higher doses of flavonoids exerts cytotoxic effects on human neuronal SH-SY5Y cells by decreasing ROS production [22]. Furthermore, fetuses exposed to a flavonoid-rich diet, especially during the third trimester of pregnancy, show higher ductal velocities, lower pulsatility indices, and larger right ventricles than those exposed to minimal amounts of these substances do [21]. Thus, in high doses, the adverse effects of flavonoids may outweigh their benefits and caution should be exercised in ingesting them at higher levels than would be obtained from a typical diet [14]. Any medication used during pregnancy, including medicinal plants, should always have its cost-effectiveness and benefit versus harm considered in every situation. The scarcity of data on the use of medication during pregnancy makes it even more critical. Several flavonoids have been shown to cross the hemato-placental barrier to accumulate in fetal tissue [23], and adaptations made by the fetus to cope with inappropriate nutrition may lead to morphological and physiological changes that persist into postnatal life [24]. Therefore, the aim of the present study was to evaluate the effect of supplementation with AS extract during pregnancy and lactation on redox parameters in Wistar rat dams and their offspring.

## 2. Experimental Section

### 2.1. Plant Material

AS inflorescences were purchased from Centro de Pesquisas Químicas, Biológicas e Agronômicas (CPQBA, Universidade de Campinas, Campinas, Brazil). The plant samples were collected, dried at room temperature in May 2013, and subsequently identified as cultivar CPQBA/2 registered at the Ministério Agricultura, Pecuária e Abastecimento (MAPA-Brazil) as number 22975.

### 2.2. Chemicals

The following chemical compounds were used: methanol (J.T., Baker, CA, USA), acetonitrile (Tedia, Aparecida de Goiânia-GO, Brazil), and phosphoric acid (Merck, Kenilworth, NJ, USA) were high-performance liquid chromatography (HPLC) grade. The standards quercetin, luteolin, and 3-O-methylquercetin were purchased from Sigma, Alfa Aesar (Karlsruhe, Germany), and Extrasynthese (Genay, France), respectively. The standard of achyrobichalcone was isolated from inflorescences of *A. satureoides* according to the method in [25].

### 2.3. Preparation of AS Extracts

Two AS extracts were prepared and the medicinal plant to solvent proportion used was 7.5:100 (*w*/*v*) for each extractive solution. The aqueous extract (AQ) was prepared by decoction, and the freeze-dried hydroalcoholic (HA) extract was prepared by macerating the inflorescences in ethanol 80% (*v*/*v*). The extraction time was eight days, and the extraction mixture was constantly stirred [4]. The extract obtained was filtered before use, frozen at −80 °C, and was subsequently dried in a freeze-dryer (Edwards Modulyo 4K, Irvine, CA, USA) at a temperature and pressure of −60 °C and −10^−2^ Bar, respectively.

### 2.4. Flavonoid Content Determination

Approximately 20 mg AS was dissolved in 20 mL 80.0% ethanol and placed in an ultrasound bath (Unique, São Paulo, Brazil) for 10 min. This solution was appropriately diluted with a methanol and 16 mM phosphoric acid solution (1:1, *v*/*v*), filtered through a 0.45-μm membrane filter (Millipore-HVHP, MA, USA), and evaluated in triplicate. Liquid chromatography (LC) analysis of the AS was carried out following a method described previously [26]. The Shimadzu LC-10A system used for the analysis was equipped with an LC-10 AD pump and a CBM-10A system controller; the system was controlled at 30 ± 1 °C, and the programmed injection volume was 20 μL. Method specificity was evaluated using a Shimadzu LC-20A system, equipped with an LC-20 AT pump, a CBM-20A system controller, an SIL-20A autosampler, and an SPD-M20A diode array detector. The limits of detection and quantitation were determined using the equations described in the International Council for Harmonization guidelines. The results are expressed as the mean of flavonoid (g) in 100 g dry extract of three analyses.

### 2.5. Animal Model and Experimental Design

The Federal University of Rio Grande do Sul Ethical Committee for Animal Experimentation reviewed and approved the study protocol (project number 21563, 19 July 2013). All experimental procedures were performed in accordance with the recommendations of the Brazilian Society for Science in Laboratory Animals. Male and female Wistar rats (90-day-old) were obtained from our breeding colony. The animals were housed in groups of four with free access to water and standard commercial food and were maintained on a 12-h light-dark cycle at a constant temperature (22 ± 4 °C) and relative humidity (30–40%). These standard conditions were maintained throughout the experiments.

**Figure 1:**
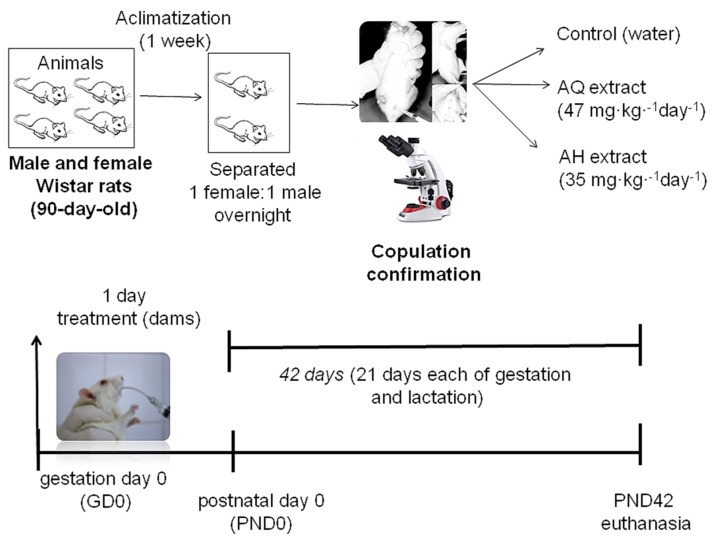
Workflow timeline and experimental design.

The pregnant rats were obtained from nulliparous females (90-day-old, weighing 200–250 g) caged with a single mature male (1 female:1 male (1F:1M)) overnight. Prior to mating, all females were checked daily for two weeks to determine their estrous cycles by direct vaginal smear examination using light microscopy and selected during the sexual receptive phase of their estrous cycles (proestrus) [27]. In the morning, the presence of a vaginal plug, viable sperm, or both in the vaginal smear was regarded as successful mating. This day was designated as gestation day 0 (GD0). The dams were allowed to litter naturally, the delivery day was defined as postnatal day 0 (PND0), and the dams were housed with their litter until euthanasia at PND42. The pregnant females were randomly divided into three groups, which were treated during pregnancy and lactation (21 days each of gestation and lactation) with of the AQ and AH extracts at concentrations equivalent to 150 mL tea from day one (47 mg·kg^−1^·day^−1^) and 35 mg·kg^−1^·day^−1^, respectively, while the control received water (Figure 1).

All female rats were observed for clinical symptoms of toxicity and mortality once a day throughout the study. The body weights of the dams were assessed on GD0, 7, 14, and 20 and lactation day (LD) 0, 7, 14, and 21, and the body weight gain was calculated. Rats that died during the administration period were necropsied and simply examined. On PND0, pups of both sexes were counted, weighed, and checked for the presence of external malformations and stillbirth. During the lactation period, the pups were examined daily for clinical signs and mortality. Litter sizes were determined on PND0; the litters were weighed on PND0, 7, 14, and 20; and the body weight gain was calculated on PND15 for eye-opening of the pups. The viability indices of the pups were calculated for each litter on PND0, 7, 14, and 21 and at the terminal necropsy; the females were confirmed for gestation by counting the number of uterine implantation sites.

### 2.6. Antioxidant Enzymes and Glutathione S-Transferase (GST)

All animals (dams and offspring rats) were euthanized by decapitation 24 h after the last extract administration, the tissues were immediately collected, and then they were frozen at −80 °C. The total protein was quantified using the Lowry assay [28] and used to normalize all the data. The catalase, superoxide dismutase (SOD), glutathione (GSH) peroxidase (GPx), and GSH S-transferase (GST) activities were quantified in the tissue homogenates of the liver, heart, kidney, cortex, hippocampus, and cerebellum of the dam and offspring rats. SOD activity was measured by quantifying the inhibition of superoxide-dependent adrenaline auto-oxidation to adrenochrome [29]. CAT activity was evaluated by following the rate of decrease in hydrogen peroxide (H_2_O_2_) absorbance at 240 nm [30]. GPx activity was measured by following the decrease of NADPH at 340 nm (37 °C) [31]. GST was measured by the to produce a colored of dinitrophenyl thioether monitored at 340 nm [32]. To better understand the effect of AS extract supplementation on these free radical-detoxifying enzymes, we determined the ratio of SOD and CAT activities (SOD/CAT), two enzymes that act in sequence to reduce the superoxide anion to water.

### 2.7. Oxidative Damage Markers

All protein oxidative damage and effects on lipids in dams and offspring rats were analyzed in tissue samples of the liver, kidney, heart, and cortex, hippocampus, and cerebellum. The oxidative status of the thiol groups was assessed by quantification of the total reduced sulfhydryl (SH) groups. Samples were reacted with 5,5′-dithionitrobis 2-nitrobenzoic acid (10 mM) during a 60-min incubation at room temperature, and the absorbance of the solution was read using a spectrophotometer at 412 nm [33]. The carbonyl groups were determined as an index of the oxidative protein damage, based on the reaction with 2,4-dinitrophenylhydrazine (DNPH), as previously described [34]. Lipoperoxidation was determined by the quantification of TBARS generated from the reaction of the thiobarbituric acid with lipoperoxides in an acid-heating medium. After precipitation with 10% trichloroacetic acid (TCA), the supernatant was mixed with 0.67% TBA and heated in a boiling water bath for 20 min. TBARS was determined by measuring the absorbance using a spectrophotometer at 532 nm [35].

### 2.8. Statistical Analysis

The statistical analysis was performed using Statistical Package for the Social Sciences software (IBM), and the results were expressed as the means ± standard error of the mean (SEM). The data were evaluated using univariate analysis of variance (ANOVA) followed by Bonferroni’s post-hoc test. Differences were considered significant when *p* < 0.05 for all the data.

## 3. Results

### 3.1. AS Extract Composition

The components of the AS HA and AQ extracts were identified using LC separation. The flavonoids quercetin, luteolin, 3-o-methylquercetin, and achyrobichalcone were the main components of both extracts, and their content in the HA (12.4 g 100 g^−1^ extract) and AQ (5.6 g 100 g^−1^ extract) are shown in Table 1. The HA contained 22.44 and 14.5% of quercetin and luteolin, respectively, which collectively corresponded to 36.9% of the total flavonoids present in the extract. The AQ contained 12.3% and 6.5% quercetin and luteolin, respectively, which constituted approximately half of the flavonoid content of the HA extract, demonstrating that ethanol had a higher extraction capacity than water did for the flavonoids in the inflorescences.

### 3.2. Reproductive, Maternal, and Litter Data in Pups

The litter sizes of the AQ and HA groups were significantly different (both *p* < 0.01) compared to that of the control group (Table 2). The change in the delivery index (relation between the number of pups delivered and the number of pups implanted multiplied by 100, *p* < 0.01 and 0.001, respectively) suggest a possible toxic effect of the AQ and HA treatment compared to the control treatment. The delivery index of the AQ group was also significantly different from that of the HA group (*p* < 0.01). During pregnancy, no differences in weight gain were observed between the groups and no malformations were observed in the pups (Table 2). The treatments did not modify the sex ratio of the litters between the groups. During lactation, the pups exhibited no intoxication symptoms related to the treatments and no treatment-induced reduction in body weights. However, we observed treatment-related differences (*p* < 0.05) in the time of eye-opening of the pups. The AQ- and HA-treated pups opened their eyes three to four days before the control group pups did (Table 2). The number of pups in the AQ group was lower (*p* < 0.05, 11 animals) than that in the control and the HA groups (24 and 20 animals, respectively), demonstrating that the AQ extract may contain teratogenic compounds.

### 3.3. Maternal Oxidative Parameters

The biochemical data showed increased SOD, CAT, and GST activities in the liver and kidneys of dams treated with AQ and HA compared with the levels in the control group (Table 3). In the heart, we observed only a significant increase in GST activity in the treated groups compared with the control group. Oxidative lipid and protein damage was determined by assaying TBARS and protein carbonylation levels, which showed no significant difference in any of the analyzed tissues. The results of tissue sample analysis of the cerebellum, frontal cortex, and hippocampus are presented in Table 3. No significant differences occurred in the enzyme activities, and no oxidative damage was observed in central nervous system (CNS) structures.

### 3.4. Pups’ Oxidative Parameters

The biochemical data of the dams showed no increases in the levels of oxidative damage markers in the studied tissues (the liver, heart, and kidney). The liver showed significant alterations in the GST, CAT, and SOD activity in the AQ and HA extract-treated groups compared to that in the control group. SOD enzymatic activity in the AQ and HA extract-treated groups were significantly higher (25.81 and 25.52 units SOD·mg^−1^ protein, respectively, *p* < 0.01) than that in the control group. In the heart tissue, no significant difference in enzymatic activities was observed (Table 4). We also analyzed the oxidative parameters in the cerebellum, frontal cortex, and hippocampus tissues, and no significant differences were observed in the enzymes’ activities, and no oxidative damage was observed in these CNS structures.

## 4. Discussion

In this study, we supplemented pregnant and lactating rats with AS extracts in doses equivalent to the consumption of 150 mL of tea per day according to the extraction yield (AQ and HA, 47 and 35 mg·kg^−1^·day^−1^, respectively). The doses used were equivalent to the mean doses ingested in tea beverages by pregnant women [13]. Equivalent doses may be obtained by applying uncertainty factors of 10-fold each for species and interspecies differences [36]. At these doses and conditions, reproductive and developmental toxicity endpoints were observed with treatment-related clinical signs of maternal and offspring toxicity in the delivery index. Furthermore, treatment-related effects, including a slight delay in the eye-opening completion rate, were found in both AS extract-treated groups. Flavonoids are extensively and rapidly metabolized by the liver by methylation, sulfonation, glucuronidation, or a combination of these processes, which likely modulates the cellular bioavailability of these compounds [37] 

Our results showed that AS extracts may induce symptoms of toxicity in dams in the delivery index; however, the exact mechanism has not yet been determined. Our results seems to be different to those of another study that reported no negative effects on fertility, fetal weight, or prenatal development when CD-1 mice were gavaged with 400 or 800 mg·kg^−1^·day^−1^ green tea extract (GTE) alone (containing epigallo-catechin-gallate (EGCG) flavonoid) from GD6 to 13 [38]. The number of pups in the AQ group was lower than that in the control and the HA groups, demonstrating that the AQ may contain compounds able to modify the oxidative biochemical parameters in dam and pups, and to have a negative impact on the delivery index and neonatal survival. Levels of enzymatic activity are considered an important factor that protects organs against the deleterious effect of potential toxicants [39], and our results showed that GST, CAT, and SOD activity in maternal livers were significantly increased. In addition, GST also detoxifies endogenous electrophiles, which are usually the consequence of free-radical damage and may be an important participant in the mechanism mediating the repair of free-radical damage [40]. Alterations in GST activity likely altered the redox state and the antioxidant defenses of the tissue [41]. Finally, GST is also an endogenous switch for the control of signaling cascade pathways, and alterations in its activity may alter the regulatory balance of numerous kinase pathways [42].

SOD is a key antioxidant enzyme implicated in the regulation of ROS-mediated tissue damage. SOD plays a key role in detoxifying superoxide anions into H_2_O_2_ and oxygen, and CAT may degrade H_2_O_2_ into water and oxygen [43]. A poor defense system allows the formation of superoxide anions and H_2_O_2_. The superoxide radical can react with NOx, generating the highly reactive peroxynitrite anion, which can induce lipid oxidation and inactivate several key SH-bearing enzymes, depleting the SH protein content [44].

GST is a detoxification enzyme [42], which acts to detoxify endogenous compounds, such as peroxidized lipids, and thereby enables the breakdown of xenobiotics. GST may also bind toxins and function as a transport protein, which explains the earlier term for GST, ligandin. These results corroborate those of another study that reported the hepatoprotective activity of achyrocline extract [45]. The major natural antioxidative components in AS extracts are flavonoids [3] and also phenolic compounds. The antioxidant activities of AS extracts have been reported by other studies and flavonoids, phenolic compounds, and achyrobichalcone were found to be the most powerful radical scavenging compounds in the extracts.

The cerebellum, frontal cortex, and hippocampus were chosen based on their critical role in the maintenance of basic brain activities. The cerebellum is thought to have a primary role in motor control and coordination, and this complex structure, which contains the majority of the brain’s neurons, has a considerable role in cognition [46]. The frontal cortex has a crucial role in brain homeostasis during adaptive behavior through its involvement in decision-making [47]. The acquisition of new memories of events and places depends on the optimal functioning of the hippocampus [48]. In humans, the development of CNS connections occurs mainly during the intrauterine phase; however, in rats, it occurs mainly during the period from the last third of the gestation until approximately the first two weeks of the suckling phase [49].

Maternal nutrition has a significant effect on developmental processes during pregnancy and lactation. Many women resort to the use of AS infusions to alleviate symptoms of pregnancy [13] and the potential health benefits are attributed to flavonoids and phenolic compounds [45,50]. The analysis of the flavonoid composition of the two extracts verified that content of the HA extract was two times that of the AQ extract based on the affinity of the compounds to the polar solvent used in the extraction [8]. This finding corroborates the result of two studies on AS freeze-dried extracts [25,26]. The rationale for focusing on the flavonoids in the extracts is the current evidence of the metabolism and transfer of flavonoids to the fetus during pregnancy. The flavanols, or more accurately their metabolites, can reach the fetal tissues, where they could potentially interact with molecules in the developmental processes [51]. Studies have reported that flavonoids, such as quercetin, induce DNA double-strand breaks and prenatal exposure to these substances slightly increases the incidence of malignancies in DNA repair-deficient mice [51]. This phenomenon is implicated in the development of cancer [21,51,52], and may pose a serious threat to the safe reproductive development. However, the evidence of the effects of AS supplementation in women during pregnancy and lactation are still limited.

Therefore, no consensus has been reached on the safety of AS supplementation during gestation in humans. Furthermore, the use of herbal medications is not strictly regulated, unlike other conventional drugs and, unfortunately, the potentially toxic effects of excessive intake are still largely ignored.

## 5. Conclusions

**Figure 2:**
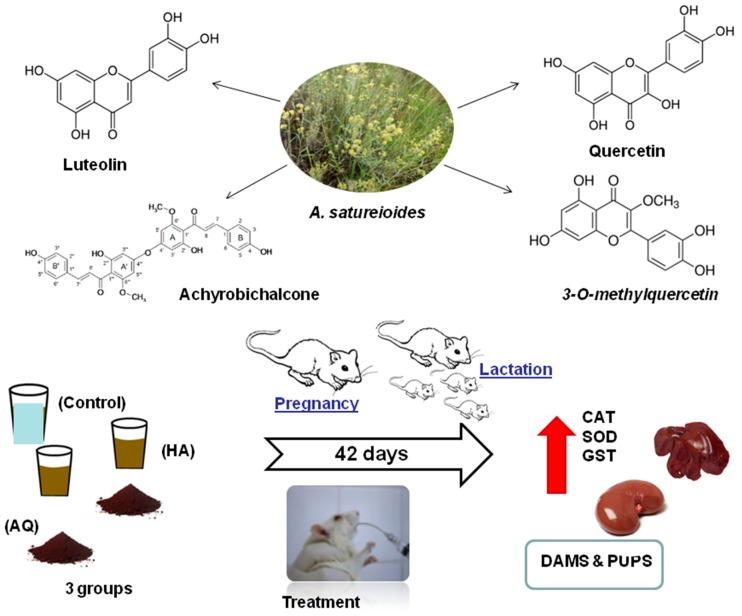
Pregnant and breastfeeding rats supplemented with *Achyrocline satureioides* inflorescence extracts showed tissue-specific changes in enzymatic activity and lower neonatal survival.

We conclude AQ in concentrations (47 mg·kg^−1^·day^−1^) of AS (35 mg·kg^−1^·day^−1^) inflorescence extracts during gestation could lead to in vivo toxicity, reflected in a decreased delivery index and neonatal survival. These effects could be related, at least in part, to variations in tissue-specific redox homeostasis and enzymatic activity, especially as the liver and kidney were affected in both dams and pups (Figure 2). It was not possible to determine whether these events could be predominantly attributed to pre- or early post-natal treatments with AQ of AS inflorescence extracts in pregnant or breastfeeding rats, respectively. This represents the greatest limitation of our study, which deserves further investigation.

## Figures and Tables

**Table 1:** Total flavonoids content of *Achyroclines satureoides* (AS) extracts (μg·mg^−1^ DW).

Samples	Quercetin (g/100 g Extract)	*3-O-methylquercetin* (g/100 g Extract)	Luteolin (g/100 g Extract)	Achyrobichalcone (g/100 g Extract)	Total Flavonoid (g/100 g Extract)
Freeze-dried hydroalcoholic	2.77 ± 0.6 ^b^	6.23 ± 0.5 ^a^	1.80 ± 0.01 ^a^	1.60 ± 0.8 ^a^	12.4 ^a^
Aqueous extract	1.68 ± 0.3 ^b^	2.70 ± 0.1 ^a^	0.50 ± 0.02 ^b^	0.60 ± 0.2 ^b^	5.6 ^b^

Data are means ± standard deviation SD. Different letters in the same column indicate significant differences in the total flavonoids content between of *Achyroclines satureoides* extracts (* *p* < 0.05). *n* = 3.

**Table 2:** Reproductive data.

*Achyrocline Satureioides* (mg·kg^−1^·day^−1^)			
Reproductive Parameters	Control (Water)	Aqueous (AQ) Extract (47)	Hydroalcoholic (HA) Extract (35)
Gestation weight gain (%)	15.8 ± 5.1	13.8 ± 7.1	14.8 ± 4.4
Lactation weight gain (%)	13.8 ± 6.1	11.5 ± 2.1	11.8 ± 2.4
Gestation length (days)	21 ± 1	23 ± 1	22 ± 1
No. of implantations	10 ± 4.1	8.5 ± 5.1	11.8 ± 2.4
Delivery index (%)	98 ± 5.1	73 ± 6.1	80 ± 5.5
Days before eye opening	13 ± 1	10 ± 2	9 ± 1*
No. of pups	24 ± 1	11 ± 6.1 *	20 ± 2.1 *^,#^
Viability index (%)			
Day 0	98.3 ± 1.6	70.3 ± 1.3 *	90.4 ± 3.6
Day 7	99.3 ± 2.1	88.2 ± 2.3 *	98.4 ± 2.9
Day 14	88.3 ± 3.1	85.3 ± 1.3	87.4 ± 3.3
Day 21	98.5 ± 1.6	97.3 ± 5.3	90.4 ± 5.6
Day 42	98.3 ± 5.6	96.3 ± 3.5	98.4 ± 3.7
Pup weight (g)			
Day 5	9.8 ± 1.1	8.2 ± 1.3	8.4 ± 1.9
Day 14	18.3 ± 2.2	15.3 ± 1.0	17.4 ± 2.4
Day 21	30.5 ± 5.2	29.3 ± 2.3	30.4 ± 2.6
Day 42	48.3 ± 3.1	44.3 ± 2.3	42.4 ± 3.7

* *p* < 0.05 and ^#^ *p* < 0.05 compared to control and AQ treatment, respectively; Gestation weight gain (%) = ((weight on PND0 − weight on GD0)/weight on GD0)) × 100. Lactation weight gain (%) = ((weight on PND21 − weight on PND0)/weight on PND0)) × 100; Delivery index (%) = (no. of pups delivered/No. of implantations) × 100; Viability index on postnatal day 0 (%) = (no. of live pups delivered/total no. of pups delivered) × 100; Viability index on postnatal day 7 (%) = (no. of live pups on postnatal day 7/no. of live pups delivered) × 100; Viability index on postnatal day 14 (%) = (no. of live pups on postnatal day 14/no. of live pups on postnatal day 7 after delivery) × 100; Viability index on postnatal day 21 (%) = (no. of live pups on postnatal day 21/no. of live pups on postnatal day 14 after delivery) × 100.

**Table 3:** Biochemical data of tissues from dams.

	*Achyrocline Satureioides* (mg·kg^−1^·day^−1^)		
Biochemical Parameters	Control (Water)	AQ (47)	HA (35)
**Liver**			
TBARS level (nmol MDA/mg protein)	7.21 ± 1.4	7.54 ± 1.1	6.11 ± 1.1
Carbonyl level (nmol carbonyl/mg protein)	1.52 ± 0.5	1.66 ± 0.5	1.71 ± 0.3
Total thiol content (mmol SH/mg protein)	52.6 ± 5.2	53.61 ± 2.8	55.51 ± 5.2
TRAP (under curve area)	354.3 ± 18.5	357.76 ± 61.1	338.7 ± 27.7
GST activity (U GST/mg protein)	0.217 ± 0.5	0.261 ± 0.3 *	0.278 ± 0.2 *
CAT activity (U CAT/mg protein)	42.6 ± 1.8	47.62 ± 2.5 *	49.79 ± 1.7 *
SOD activity (U SOD/mg protein)	32.76 ± 9.4	38.32 ± 1.5 *	39.09 ± 2.5 *
SOD/CAT ratio (arbitrary units)	0.76 ± 0.3	0.80 ± 0.5 *	0.78 ± 0.9 *
**Kidney**			
TBARS level (nmol MDA/mg protein)	5.34 ± 1.8	3.28 ± 1.6	4.3846 ± 1.5
Carbonyl level (nmol carbonyl/mg protein)	1.77 ± 0.2	1.73 ± 0.5	1.38 ± 0.3
Total thiol content (mmol SH/mg protein)	44.96 ± 5.9	45.53 ± 6.2	47.34 ± 5.3
TRAP (under curve area)	176.3 ± 27.8	224.3 ± 22.7	178.8 ± 21.79
CAT activity (U CAT/mg protein)	31.87 ± 4.1	37.48 ± 3.1 *	36.43 ± 3.4 *
SOD activity (U SOD/mg protein)	18.16 ± 2.5	19.81 ± 6.5 *	20.52 ± 1.2 *
GST activity (U GST/mg protein)	0.217 ± 0.5	0.251 ± 0.3 *	0.248 ± 0.2 *
SOD/CAT ratio (arbitrary units)	0.56 ± 0.6	0.52 ± 0.7 *	0.56 ± 0.7
**Heart**			
TBARS level (nmol MDA/mg protein)	2.0379 ± 0.2	2.2703 ± 0.5	2.1881 ± 0.6
Carbonyl level (nmol carbonyl/mg protein)	2.0249 ± 0.3	1.9941 ± 0.2	1.6428 ± 0.4
Total thiol content (mmol SH/mg protein)	35.01 ± 5.8	36.91 ± 8.5	43.95 ± 7.4
TRAP (under curve area)	488.45 ± 48.0	401.53 ± 22.55	407.7 ± 60.39
CAT activity (U CAT/mg protein)	6.42 ± 0.5	6.43 ± 1.6	6.3 ± 2.0
SOD activity (U SOD/mg protein)	5.25 ± 1.2	6.75 ± 2.7	6.5 ± 1.7
GST activity (U GST/mg protein)	0.217 ± 0.8	0.251 ± 0.2 *	0.248 ± 0.3 *
SOD/CAT ratio (arbitrary units)	0.81 ± 0.8	1.04 ± 0.8 *	1.03 ± 0.8 *
**Cerebellum**			
TBARS level (nmol MDA/mg protein)	6.81 ± 5.4	6.54 ± 1.6	6.01 ± 1.1
Carbonyl level (nmol carbonyl/mg protein)	1.2 ± 0.3	1.6 ± 0.9	1.0 ± 0.7
Total thiol content (mmol SH/mg protein)	50.6 ± 2.2	52.61 ± 8.8	49.51 ± 7.2
CAT activity (U CAT/mg protein)	4.66 ± 1.8	4.62 ± 3.5	6.79 ± 1.7
SOD activity (U SOD/mg protein)	6.76 ± 1.4	10.32 ± 4 *	11.09 ± 2.5
SOD/CAT ratio (arbitrary units)	1.45 ± 1.3	2.23 ± 0.8 *	1.63 ± 0.9
**Hippocampus**			
TBARS level (nmol MDA/mg protein)	5.38 ± 1.8	3.08 ± 1.6	4.46 ± 1.5
Carbonyl level (nmol carbonyl/mg protein)	1.77 ± 0.2	1.76 ± 0.5	1.28 ± 0.3
Total thiol content (mmol SH/mg protein)	44.96 ± 5.9	45.53 ± 6.2	47.34 ± 5.3
CAT activity (U CAT/mg protein)	11.87 ± 4.1	12.48 ± 2.1	10.43 ± 3.4
SOD activity (U SOD/mg protein)	8.16 ± 2.5	5.81 ± 1.5 *	5.52 ± 3.2 *
SOD/CAT ratio (arbitrary units)	0.68 ± 0.1	0.46 ± 0.1 *	0.52 ± 0.2
**Cortex**			
TBARS level (nmol MDA/mg protein)	2.0 ± 0.2	2.03 ± 0.5	2.81 ± 0.6
Carbonyl level (nmol carbonyl/mg protein)	2.49 ± 0.3	1.99 ± 0.2	1.68 ± 0.4
Total thiol content (mmol SH/mg protein)	35.01 ± 5.8	36.91 ± 8.5	43.95 ± 7.4
CAT activity (U CAT/mg protein)	6.42 ± 0.5	6.43 ± 1.6	6.51 ± 0.2
SOD activity (U SOD/mg protein)	5.25 ± 1.2	6.75 ± 2.7	6.31 ± 0.8
SOD/CAT ratio (arbitrary units)	0.81 ± 1.8	1.04 ± 2.8	0.96 ± 1

* Significantly different from control; *n* = 6. TBARS, thiobarbituric reactive species; TRAP, total reactive antioxidant potential; GST, glutathione *S*-transferase; CAT, catalase; SOD, superoxide dismutase; MDA, malondialdehyde.

**Table 4:** Biochemical data of the tissues of the pups.

*Achyrocline satureioides* (AS, mg·kg^−1^·day^−1^)			
	**Control (Water)**	**AQ (47)**	**HA (35)**
**No. of Pups Examined**	24	11	20
**Liver**			
TBARS level (nmol MDA/mg protein)	6.81 ± 1.4	6.54 ± 1.6	6.01 ± 1.1
Carbonyl level (nmol carbonyl/mg protein)	1.62 ± 0.3	1.66 ± 0.8	1.80 ± 0.7
Total thiol content (mmol SH/mg protein)	55.6 ± 2.2	58.61 ± 8.8	53.51 ± 6.2
TRAP (under curve area)	324.3 ± 55.25	317.76 ± 29.9	318.37 ± 7.1
CAT activity (U CAT/mg protein)	42.66 ± 1.8	54.62 ± 3.5 *	56.79 ± 1.7 *
SOD activity (U SOD/mg protein)	36.76 ± 1.4	40.32 ± 1.5 *	46.09 ± 2.5 *
SOD/CAT ratio (arbitrary units)			
GST activity (U GST/mg protein)	0.217 ± 0.5	0.251 ± 0.3 *	0.248 ± 0.2 *
**Kidney**			
TBARS level (nmol MDA/mg protein)	5.38 ± 1.8	3.08 ± 1.6	4.46 ± 1.5
Carbonyl level (nmol carbonyl/mg protein)	1.77 ± 0.2	1.76 ± 0.5	1.28 ± 0.3
Total thiol content (mmol SH/mg protein)	44.96 ± 5.9	45.53 ± 6.2	47.34 ± 5.3
TRAP (under curve area)	176.6 ± 27.82	124.33 ± 32.76	128.78 ± 21.79
CAT activity (U CAT/mg protein)	31.87 ± 4.1	37.48 ± 3.1	36.43 ± 3.4
SOD activity (U SOD/mg protein)	18.16 ± 2.5	25.81 ± 1.5 *	25.52 ± 3.2 *
SOD/CAT ratio (arbitrary units)	0.56 ± 0.5	0.68 ± 0.2	0.70 ± 1.0
GST activity (U GST/mg protein)	0.05 ± 0.7	0.08 ± 0.5	0.09 ± 0.6
**Heart**			
TBARS level (nmol MDA/mg protein)	2.0 ± 0.2	2.03 ± 0.5	2.81 ± 0.6
Carbonyl level (nmol carbonyl/mg protein)	2.49 ± 0.3	1.99 ± 0.2	1.68 ± 0.4
Total thiol content (mmol SH/mg protein)	35.01 ± 5.8	36.91 ± 8.5	43.95 ± 7.4
TRAP (under curve area)	488.5 ± 48.57	401.5 ± 22.57	407.89 ± 60.4
CAT activity (U CAT/mg protein)	6.42 ± 0.5	6.43 ± 1.6	6.5 ± 1.3
SOD activity (U SOD/mg protein)	5.25 ± 1.2	6.75 ± 2.7	6.3 ± 2.3
SOD/CAT ratio (arbitrary units)	0.81 ± 1.7	1.04 ± 1.2	0.96 ± 2.9
GST activity (U GST/mg protein)	0.06 ± 0.7	0.05 ± 0.7	0.06 ± 0.5
**Cerebellum**			
TBARS level (nmol MDA/mg protein)	6.81 ± 1.4	6.54 ± 1.6	6.01 ± 1.1
Carbonyl (nmol carbonyl/mg protein)	1.62 ± 0.3	1.66 ± 0.8	1.80 ± 0.7
Total thiol (mmol SH/mg protein)	55.6 ± 2.2	58.61 ± 8.8	53.51 ± 6.2
CAT activity (U CAT/mg protein)	2.66 ± 1.8	4.2 ± 3.5	6.79 ± 1.7
SOD activity (U SOD/mg protein)	36.76 ± 1.4	40.2 ± 1.5	46.09 ± 2.5
SOD/CAT ratio (arbitrary units)	0.86 ± 1.8	0.74 ± 1.2	0.81 ± 1.6
**Hippocampus**			
TBARS level (nmol MDA/mg protein)	3.38 ± 1.8	3.08 ± 1.6	4.46 ± 1.5
Carbonyl (nmol carbonyl/mg protein)	1.77 ± 0.2	1.76 ± 0.5	1.28 ± 0.3
Total thiol (mmol SH/mg protein)	44.96 ± 5.9	45.53 ± 6.2	47.34 ± 5.3
CAT activity (U CAT/mg protein)	4.87 ± 4.1	7.48 ± 5.1	6.43 ± 3.4
SOD activity (U SOD/mg protein)	5.16 ± 2.5	5.81 ± 5.5	5.52 ± 3.2
SOD/CAT ratio (arbitrary units)	1.05 ± 1.5	0.77 ± 2.3	0.85 ± 0.5
**Cortex**			
TBARS level (nmol MDA/mg protein)	2.0 ± 0.2	2.03 ± 0.5	2.81 ± 0.6
Carbonyl (nmol carbonyl/mg protein)	2.49 ± 0.3	1.99 ± 0.2	1.68 ± 0.4
Total thiol (mmol SH/mg protein)	15.01 ± 5.8	16.91 ± 8.5	13.95 ± 7.4
CAT activity (U CAT/mg protein)	3.2 ± 0.5	3.43 ± 1.6	3.5 ± 1
SOD activity (U SOD/mg protein)	3.25 ± 1.2	3.75 ± 2.7	3.31 ± 2
SOD/CAT ratio (arbitrary units)	1.01± 3.5	1.09 ± 2.2	0.94 ±3.6

* Significantly different from the control. TBARS, thiobarbituric reactive species; TRAP, total reactive antioxidant potential; GST, glutathione *S*-transferase; CAT, catalase; SOD, superoxide dismutase; MDA, malondialdehyde.
